# Urogenital Schistosomiasis on Santiago Island, Cabo Verde, 2023

**DOI:** 10.1590/0037-8682-0318-2025

**Published:** 2025-12-15

**Authors:** Ludmila Miranda, Sarah Mendes D’Angelo, Liliane Hungria, Menilita Santos, Admilson Ramos de Oliveira, Veruska Maia Costa, Silvinia Duarte, Giovanni Veríssimo Lima, Lauro Perdigão, Maria Ilisita Ramos Fernandes Fonseca, Valdir Correia Rodrigues, Águida Madalena Ribeiro Semedo, Janilza Silveira Silva, Angelo de Jesus Cunha Cardoso, Janice de Jesus Xavier Soares, Jaelsa Moreira, Ivaldina Vaz, Maria da Luz Lima Mendonça, Luciano Pamplona de Góes Cavalcanti

**Affiliations:** 1 Ministério da Saúde de Cabo Verde, Cabo Verde, África.; 2 Escola de Saúde Pública do Ceará, Fortaleza, CE, Brasil.; 3 Instituto Nacional de Saúde Pública de Cabo Verde, Cabo Verde, África.; 4 Fundação Oswaldo Cruz, Brasília, DF, Brasil.; 5 Serviço Nacional de Proteção Civil e Bombeiros de Cabo Verde, Cabo Verde, África.; 6 Secretaria de Saúde do Ceará, Fortaleza, CE, Brasil.; 7 Universidade Federal do Ceará, Programa de Pós-Graduação em Saúde Pública, Fortaleza, CE, Brasil.; 8 Ministério da Agricultura e Meio Ambiente de Cabo Verde, Cabo Verde, África.; 9 Centro Universitário Christus, Faculdade de Medicina, Fortaleza, CE, Brasil.

**Keywords:** Urogenital schistosomiasis, Schistosoma haematobium, Schistosomiasis, Neglected tropical diseases, Cabo Verde

## Abstract

**Background:**

Schistosomiasis, caused by *Schistosoma haematobium*, is a neglected disease that affects millions of people, primarily in developing countries.

**Methods:**

We describe cases of urogenital schistosomiasis that occurred in Cabo Verde between 2022 and 2023 along with their possible associated risk factors. This analytical, cross-sectional study included 229 suspected cases.

**Results:**

Of 229 cases, 53 (23.1%) were confirmed. The most affected age group was 15-24 years, with an incidence rate of 12 cases per 1,000 inhabitants. Male patients were more affected, with a male-to-female ratio of 12:1. The most common symptoms were hematuria (58.7%) and dysuria (17.3%). The incidence of the disease was 6.8 times higher among individuals who had contact with water from tanks.

**Conclusion:**

This study revealed the emergence of urogenital schistosomiasis in Cabo Verde, highlighting the need to strengthen surveillance, prevention, and control measures.

## INTRODUCTION

Schistosomiasis is a parasitic disease caused by trematodes of the *Schistosoma* genus that affects millions of people worldwide. In 2019, the disease was reported in 78 countries, with an estimated 236.6 million individuals in need of preventive treatment globally^1^.

Among various *Schistosoma* species, *Schistosoma haematobium* is responsible for urogenital schistosomiasis, a particularly debilitating form of the disease^2^. This persistent condition remains a major public health challenge, particularly in low-income settings with limited access to safe water, adequate sanitation, and essential diagnostic and treatment services^1^ ^,^ ^3^ ^,^ ^4^.

Complications associated with urogenital schistosomiasis, such as hematuria, abdominal pain, and genital itching, can significantly impair quality of life and may lead to severe health consequences^2^ ^,^ ^5^.

Cabo Verde, an archipelago off the western coast of Africa, is not exempt from the burden of neglected tropical diseases. The first investigation of human cases of urogenital schistosomiasis in the country began in 2021/2022, when eight confirmed cases were reported in the municipality of São Miguel, located in the interior of Santiago Island^6^. This investigation was conducted as a part of field activities under the Cabo Verde Field Epidemiology Training Program (EpiCV).

Considering this context, the present study aimed to analyze cases of urogenital schistosomiasis reported in São Miguel, Santiago Island, between October 2022 and July 2023. This study also describes the outbreak dynamics, explores potential risk factors associated with *S. haematobium* infection, and outlines recommendations for targeted prevention and control measures.

## METHODS

### Study Design

This cross-sectional analytical study was conducted in the municipality of São Miguel on Santiago Island, Cabo Verde. Data were collected between July and August 2023, based on patient admission records from the São Miguel Primary Health Care Center. Home visits were conducted between October 2022 and August 2023 to complement the collected information.

### Study Setting

The study was conducted in the municipality of São Miguel, Santiago Island, Cabo Verde. The municipality covers an area of 90.2 km² and has a population of 12,996. It comprises 23 localities and borders the municipalities of Santa Cruz, Santa Catarina, and Tarrafal. The economy of São Miguel is primarily based on livestock, agriculture, and fishing^7^.

### Case Definitions

The following case definitions were established for this study:

**Suspected Case**: Any individual presenting with dysuria, with or without hematuria, pruritus, and/or abdominal pain who sought care at the São Miguel Primary Health Care Center during the study period.

**Laboratory-Confirmed Case**: A suspected case with a positive laboratory diagnosis of *Schistosoma haematobium* based on urine or biopsy samples.

**Clinically-Epidemiologically Confirmed Case**: A suspected case in which no laboratory samples were collected, but whose symptoms resolved after specific treatment for *S. haematobium*.

**Probable Case**: An individual who met the definition of a suspected case but was neither confirmed nor ruled out by laboratory or clinical criteria.

### Eligibility Criteria

Pregnant women and individuals who could not be reached by phone, despite multiple attempts on different days and at various times, were excluded from the study. In addition, suspected cases that tested negative for *S. haematobium* in both urine and biopsy samples, as well as those who did not undergo sample collection and whose symptoms resolved after non-specific treatment, were excluded.

### Data Collection procedures

During home visits, blood and urine samples were collected, and a semi-structured questionnaire was administered. Trained personnel also conducted environmental inspections around the households to search for and collect snails. In some cases, follow-up telephone interviews were conducted to clarify the responses or complete missing information.

### Data Analysis

Blood and urine samples were sent to Santa Rita Vieira Regional Hospital for laboratory analysis. Snail specimens from the *Bulinus* genus were forwarded to the National Institute of Public Health (INSP) laboratory, where real-time polymerase chain reaction (RT-PCR) tests were performed for molecular characterization. All data collected from patient admission forms and questionnaires were entered into a Microsoft Excel (2019) database and subsequently analyzed using ArcGIS Survey version 10.8.

The variables used in this study were demographic data including sex, age, signs, and symptoms. We also investigated swimming habits in water tanks and works with agriculture. The positivity for urogenital schistosomiasis was evaluated. The association between the frequency of urogenital schistosomiasis and other variables was checked using Pearson’s chi-square or Fisher’s exact tests. Adjusted odds ratios (ORs) and 95% confidence intervals (CIs) were calculated to determine factors independently associated with urogenital schistosomiasis positivity. All test results were considered statistically significant at p< 0.05.

### Spatial Analysis

Mapping of water tanks containing snails and the geographic distribution of confirmed cases were conducted using ArcMap in the ArcGIS software suite.

### Ethical Considerations

All investigated patients consented to participate in the study through a Free and Informed Consent Form (FICF), and in the case of minors, consent was provided by their legal guardians. This study was authorized by the National Institute of Public Health of Cabo Verde (INSP) as part of a field epidemiology training program. 

## RESULTS

In total, 229 suspected cases were identified. Of these, 113 (49.3%) were excluded based on clinical criteria, 60 (26.2%) were classified as probable, 53 (23.1%) were confirmed by clinical or laboratory criteria, and 3 (1.3%) were excluded based on negative laboratory results. Among the confirmed cases, males were predominant (n = 49, 92.5%), with a male-to-female ratio of 12:1. The mean age was 19 years, with a median age of 16 years (range, 7-70 years) and a modal age of 17. Most cases occurred among youth aged 15-24 years (Table 1), peaking in January 2023 (n = 8; 42.1%). The highest incidence was observed in the 15-24 years age group, with 12.1 cases per 1000 inhabitants (Table 1).

**TABLE 1:** Confirmed cases and incidence of urogenital schistosomiasis by sex and age group, Santiago Island, Cabo Verde, 2022-2023.

Age group	M	%	F	%	Total	Population	**Incidence rate *(per 1,000 inhabitants)***
0 - 4	0	0.0	0	0.0	0	1,310	0.0
5 - 14	17	34.7	2	50.0	19	2,606	7.3
15 - 24	27	55.1	1	25.0	28	2,316	12.1
25 - 44	3	6.1	0	0.0	3	3,375	1.2
45 - 64	1	2.0	1	25.0	2	2,143	0.5
65 - 70	1	2.0	0	0.0	1	527	1.9
**Total**	**49**	**100.0**	**4**	**100.0**	**53**	**12,277**	**4.3**

**Footnote:** M: male; F: female.

The most frequently reported symptom was hematuria, which was present in 58.7% of confirmed cases (Table 2). The most commonly affected individuals were students (75.6%) and farmers (19.5%). Home visits accounted for 58.5% of the care provided to confirmed cases. Only 37.7% of the patients sought care at a local health center and 3.8% sought hospital-based care.

**TABLE 2:** Signs and symptoms of confirmed cases of urogenital schistosomiasis, Santiago Island, Cabo Verde, 2022-2023.

Signs and symptoms	N	%	95%CI
Hematuria	31	58.7	44.1 - 71.9
Fever	29	54.7	40.4 - 68.4
Chills	23	43.4	29.8 - 57.7
Dysuria	13	24.5	13.8 - 38.3
Body pain / Myalgia	13	24.5	13.8 - 38.3
Headache	12	22.6	12.3 - 36.2
Diarrhea	11	20.8	10.8 - 34.1
Genital pruritus	10	18.9	9.4 - 32.0
Nausea	10	18.9	9.4 - 32.0
Abdominal pain	8	15.1	6.7 - 27.6
Vomiting	8	15.1	6.7 - 27.6
Cutaneous lesions	3	5.7	1.2 - 15.7

Footnote: n=53 confirmed cases.

The risk of infection was 6.8 times higher among individuals who reported exposure to water from local tanks than among those who did not. The chi-square test results indicated a significant association between sociodemographic variables and confirmed *S. haematobium* infections (p < 0.05) (Table 3).

Males were 27 times more likely to be infected with *S. haematobium* compared to females (p < 0.001). Individuals aged 15-24 and 25-44 years were six and eight times more likely to be infected, respectively, than those aged 45-70 years (p = 0.006). Those who swam in water tanks had a 14-fold increased risk of infection compared with those who did not (p = 0.001). Farmers were four times more likely to be infected with *S. haematobium* than non-farmers (p = 0.016) (Table 3).

**TABLE 3:** Analysis of the odds of infection according to sociodemographic variables and leisure habits among cases of urogenital schistosomiasis, Cabo Verde, 2022-2023.

Variables	***S. haematobium* n (%)**		OR	95% CI	p-value
	Confirmed	Not confirmed			
Sex					
Male	49 (47.1)	55 (52.9)	26.95	(9.264 - 78.403)	<0.001
Female	4 (3.2)	121 (96.8)	1		
Age group					
0 - 14	19 (32.2)	40 (67.8)	6.17	(1.326 - 28.758)	0.020
15 - 24	28 (39.4)	43 (60.6)	8.46	(1.861 - 38.509)	0.006
25 - 44	4 (5.6)	67 (94.4)	0.78	(0.134 - 4.497)	0.777
45 - 64	2 (7.1)	26 (92.9)	1		
Swims in water tanks					
Yes	31 (64.6)	17 (35.4)	13.68	(2.790 - 67.037)	0.001
No	2 (11.8)	15 (88.2)	1		
Farmer					
Yes	8 (57.1)	6 (42.9)	4	(1.298 - 12.327)	0.016
No	35 (25.0)	105 (75.0)	1		

**FIGURE 1:**
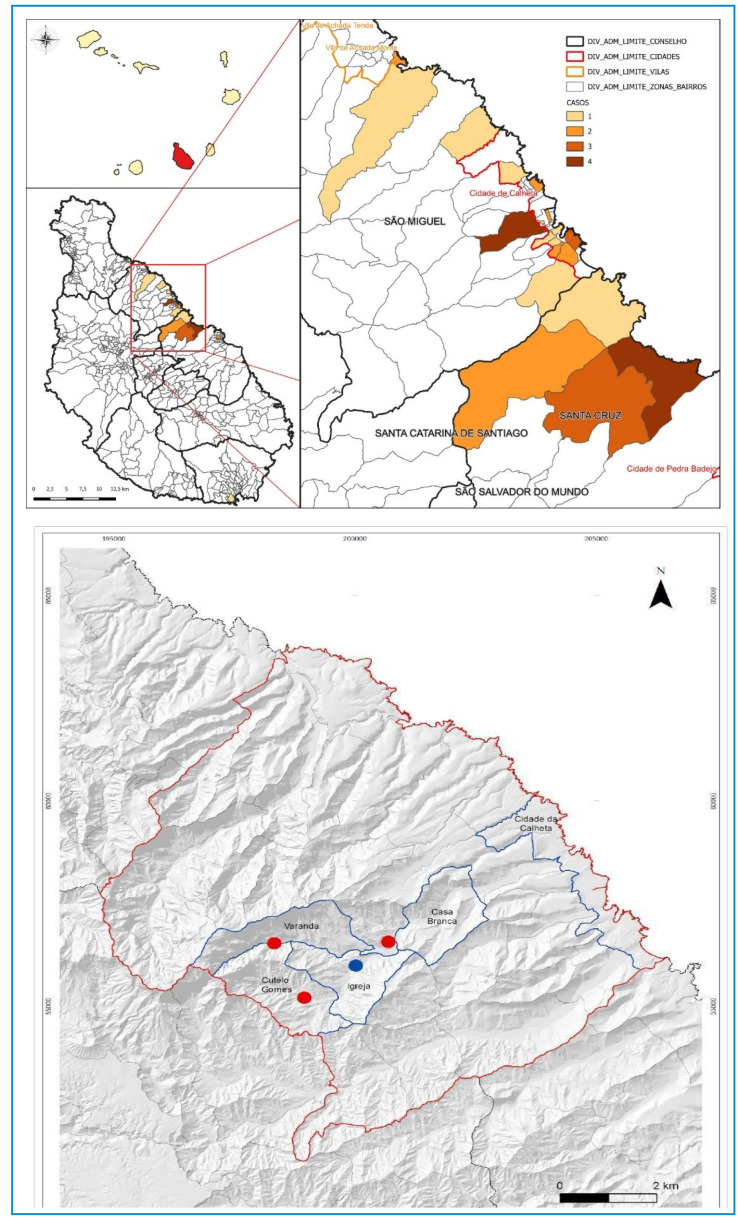
Confirmed cases of urogenital schistosomiasis by place of residence and distribution of water tanks by locality, Santiago Island, Cabo Verde, 2022-2023.

The highest numbers of confirmed cases were recorded in Cutelo Gomes and São Miguel (Varanda), accounting for 54.7% and 26.4% of the cases, respectively, which coincided with the location of the water tanks (Figure 1).

## DISCUSSION

Upon analysis, we found that urogenital schistosomiasis predominantly affected young males, and exposure to water tanks was identified as the main risk factor for infection.

This study found a high incidence rate in the 15-24 year age group (12 cases per 1000 inhabitants), aligning with patterns observed in other regions^8^ ^,^ ^9^. While some studies have reported a higher incidence among school-aged children, this may be attributed to their school-based sampling approach, which differs from our community-based investigation^10^ ^-^ ^12^. These findings underscore the importance of developing targeted control strategies in high-risk populations.

The marked predominance of male cases (12:1) highlights the role of sex in urogenital schistosomiasis prevalence. This sex disparity is commonly reported in endemic regions and can be attributed, in part, to greater male participation in water-related recreational activities^4^. Furthermore, our findings are consistent with those of previous studies emphasizing that recreational water exposure is a significant risk factor for *S. haematobium* infection^9^ ^,^ ^10^ ^,^ ^13^.

Regarding clinical manifestations, hematuria was highly frequent, reported in 58.7% of the confirmed cases. This reflects the severity of urinary tract involvement and highlights the importance of early diagnosis and appropriate treatment to prevent complications, such as bladder fibrosis and bladder cancer. The symptoms reported by the participants in this study, including hematuria, dysuria, genital itching, and abdominal pain, were consistent with the typical clinical presentation of urogenital schistosomiasis^4^ ^,^ ^5^.

An association between the presence of water tanks and the occurrence of urogenital schistosomiasis was evident in this study, with individuals exposed to these environments having a 6.8-fold greater risk of infection. This finding supports the hypothesis that contaminated water is the major vehicle for *S. haematobium* transmission^8^ ^,^ ^14^. Communities that rely on tanks or natural water bodies for daily use or recreation are at increased risk and should, therefore, be prioritized for preventive and control interventions^15^.

The results highlight the need for an integrated surveillance and control approach, including active case finding and early diagnosis, to interrupt disease transmission and prevent severe outcomes. Interventions such as covering water tanks, routinely monitoring water quality, including testing for snail presence, and implementing snail control measures are necessary to reduce the impact of urogenital schistosomiasis and improve the health of at-risk communities^15^. Some authors suggest the mass administration of praziquantel to transition from control to elimination of schistosomiasis, especially in areas of low infection intensity and for individuals with mild infections^16^.

These findings contribute to a clearer understanding of urogenital schistosomiasis in Cabo Verde, reinforcing its relevance within the broader context of neglected tropical diseases, and emphasizing the need for integrated strategies encompassing prevention, diagnosis, and treatment. The knowledge generated in this study can inform public health policies aimed at eliminating urogenital schistosomiasis in Cabo Verde and other regions facing similar health challenges. Furthermore, other studies have shown the potential risk of outbreaks in other European areas, warranting close monitoring and surveillance of all potential transmission foci^17^.

This study has some limitations that must be acknowledged. The study relied on retrospective data from previously attended patients, which were supplemented by interviews. Despite logistical efforts, the study area did not include remote or hard-to-reach zones, which may have affected the completeness of the data collection. Additionally, logistical challenges during fieldwork have hindered the proper identification of snail specimens.

## Data Availability

Research data is only available upon request.
